# Significant alterations in peripheral lymphocyte subsets and immune-related protein profiles in patients with periprosthetic joint infection

**DOI:** 10.3389/fimmu.2025.1648150

**Published:** 2025-11-19

**Authors:** Zhuo Li, Fan Yang, Jun Fu, Zhi-Yuan Li, Li-Bo Hao, Lin Yuan, Ji-Ying Chen, Chi Xu

**Affiliations:** 1 School of Medicine, Nankai University, Tianjin, China; 2 Department of Joint Surgery, Shandong Provincial Hospital Affiliated to Shandong First Medical University, Jinan, China; 3 Department of Joint Surgery, The First Medical Center, Chinese People’s Liberation Army of China (PLA) General Hospital, Jinan, China; 4 Department of Joint Surgery, The Fourth Medical Center, Chinese PLA General Hospital, Beijing, China

**Keywords:** periprosthetic joint infection, lymphocyte subsets, immune-related protein, diagnosis, haptoglobin

## Abstract

**Purpose:**

While local immune responses in periprosthetic joint infection (PJI) are increasingly studied, systemic immune alterations remain poorly characterized. Therefore, this study aimed to investigate the change in peripheral lymphocyte subsets and immune-related protein profiles in patients with PJI, and explore the potential value of these indicators for the diagnosis of PJI.

**Methods:**

Between July 2023 and January 2024, this prospective study recruited 82 patients who had been diagnosed with PJI or aseptic failure (AF), or who were healthy controls. Peripheral blood lymphocyte subpopulations and immune-related proteins were measured using flow cytometry or nephelometry and compared between groups. The diagnostic capability of different indicators for PJI was assessed. Besides, candidate markers were validated in an independent prospective cohort.

**Results:**

Compared with the AF group, the proportion and absolute counts of natural killer (NK) cells in the PJI group were significantly higher, while those of B cells were lower. Differences in most immune-related proteins were observed between PJI and AF cases. Of them, the haptoglobin was the most notably increased in the PJI group than in the AF group (245.08 ± 99.00 mg/dl vs. 108.22 ± 52.37 mg/dl, *P* < 0.001), which exhibited the best diagnostic performance with an area under the curve (AUC) of 0.890.(95% CI, 0.803-0.978). When haptoglobin was combined with C-reactive protein (CRP), the AUC for the diagnosis of PJI increased to 0.937 (95% CI, 0.876-0.998). No significant differences were observed between the AF and primary total joint arthroplasty (TJA) groups regarding these immune-related indicators. In addition, the diagnostic efficacy of haptoglobin was validated in an independent cohort.

**Conclusions:**

The systemic immune dysregulation observed in PJI patients can lay the foundation for further in-depth understanding of the immune response in PJI. The immune-related markers demonstrated promising value in diagnosing PJI, especially when synovial fluid was unavailable. Multicenter validation was warranted to confirm clinical utility.

## Introduction

Periprosthetic joint infection (PJI), while relatively uncommon in primary arthroplasty (typically 1-2%), is a devastating complication following total joint arthroplasty (TJA) that places a significant economic burden on healthcare systems worldwide and is expected to increase in the future (1, 2). Its incidence rises dramatically after revision surgery, and the absolute number of patients affected globally is substantial (3). Despite substantial research into the diagnostics and treatment strategies for PJI, there have been no significant improvements in therapeutic outcomes over the past two decades (4). This stagnation has spurred an increased scholarly focus on understanding the pathophysiological mechanisms underpinning PJI, evidenced by a thirtyfold increase in related publications over the last 20 years (5).

Recent scholarly attention has gravitated towards the role of the immune system in infection defense, prompting investigations into novel biomarkers (6–8). Korn et al. (9) employed high-dimensional flow cytometry to analyze the immune cell composition of synovial fluid after knee or hip arthroplasty, highlighting its potential as an effective screening tool for PJI. Notably, differences in the levels of soluble immune checkpoint molecules (such as sBTLA, sCD28, and sCTLA-4) between patients with PJI and those experiencing aseptic failure have been identified, suggesting novel targets for PJI diagnosis and therapy (10). Moreover, Fisher et al. (11) have pioneered the use of RNA sequencing and deconvolution techniques to delineate the immune cell spectrum within synovial fluid, introducing innovative avenues for PJI diagnostics.

However, the majority of these studies have concentrated predominantly on the localized immune responses at the infection sites, with insufficient exploration into the systemic immune responses induced by implant infections and their roles in eradicating local infections (12). Recent research highlighted the complex interaction between systemic and local immune environments, with local implant materials potentially altering systemic immune equilibrium to facilitate tissue remodeling (13). Additionally, synovial fluid-based markers for the diagnosis of PJI are not always available as joint aspiration is an invasive test, and a “dry tap” may occur in almost half of joint aspirations while radiological guidance was used (14). Therefore, there is an urgent need to develop reliable, cost-efficient analytical methods for detecting blood-based biomarkers to facilitate PJI diagnosis. These facts further underscore the necessity for a comprehensive understanding of the systemic immune status in PJI patients.

A growing body of evidence from other infectious diseases, such as sepsis, has highlighted the pivotal role of lymphocyte subsets in maintaining immune homeostasis. This could be supported by the correlation between peripheral blood lymphocyte profiles and sepsis prognosis (15, 16). However, the characterization of peripheral blood lymphocyte subsets in PJI patients remains poorly defined. Furthermore, the serum immune-related proteins, including complement, immunoglobulins (Ig), and haptoglobin, have not been comprehensively investigated in PJI patients. This knowledge gap hindered our understanding of systemic immune response alterations and impedes the development of novel diagnostic and therapeutic markers for PJI.

Therefore, this study aimed to: (1) investigate the change in peripheral lymphocyte subsets and immune-related protein profiles in patients with PJI, and (2) explore the potential value of these indicators for the diagnosis of PJI.

## Patients and methods

### Study design and settings

This prospective study, approved by the Institutional Ethics Committee, included the patients scheduled for hip or knee revision arthroplasty from June 2023 to January 2024 at our department. Inclusion criteria were: (1) adults aged between 18 and 80 years; (2) those scheduled for hip or knee revision surgery; (3) those with complete preoperative medical records and available peripheral blood samples; (4) those who provided informed consent. Exclusion criteria were: (1) patients with infections at other sites within two weeks, such as respiratory or urinary tract infections; (2) those with a history of malignant tumors; (3) those with autoimmune diseases such as systemic lupus erythematosus, psoriasis, or ankylosing spondylitis, or recent use of immunomodulatory drugs; (4) those with recent extensive skin ulceration, severe hematoma, or traumatic fracture; (5)those with acute PJI occurring within three months of TJA. PJI was diagnosed using the Musculoskeletal Infection Society (MSIS) criteria (17).

### Participants

**Figure 1:**
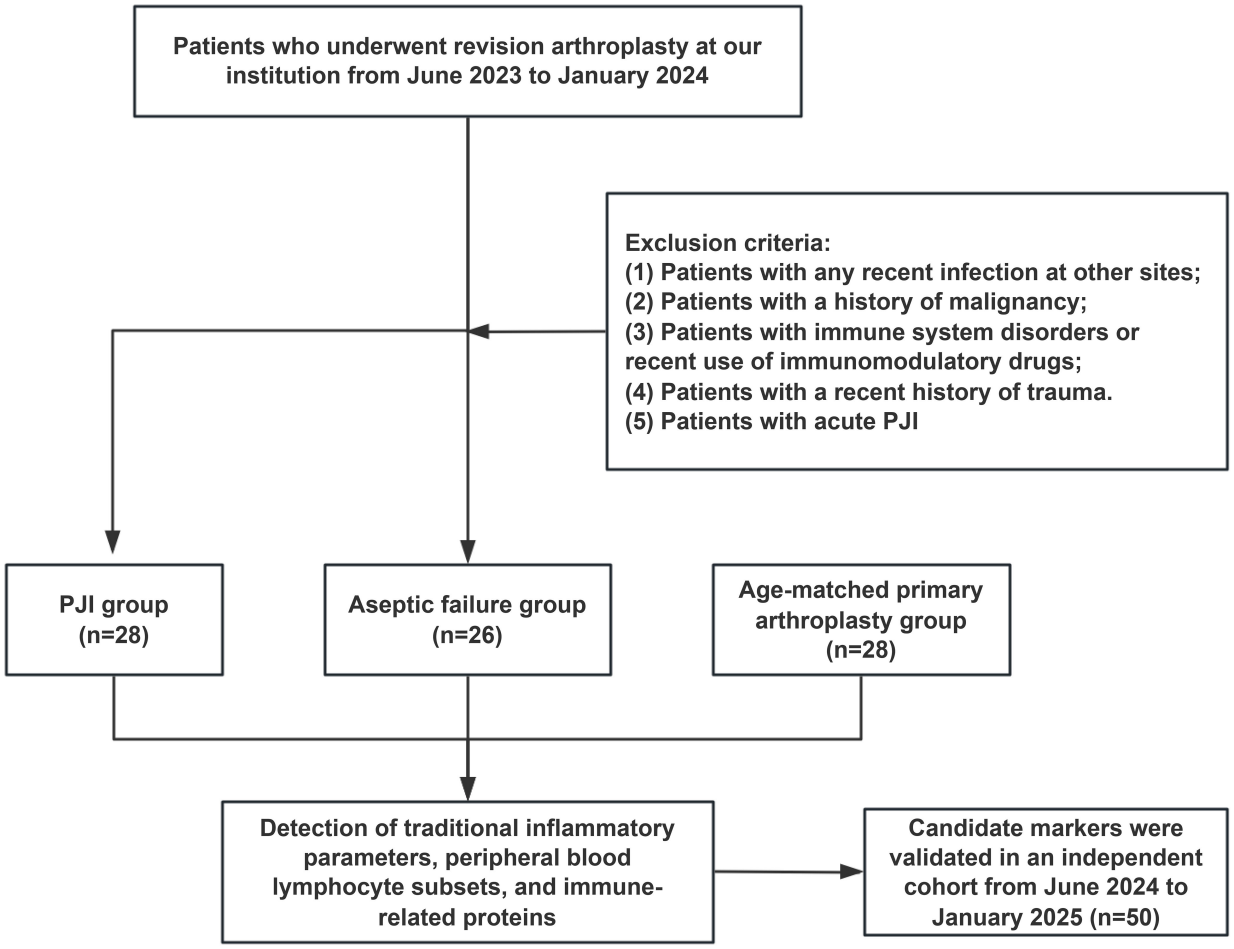
Flowchart of the inclusion of patients in this study.

The study initialy included 54 patients who had undergone revision surgery, of whom 28 were diagnosed with PJI and 26 with aseptic failure (AF). Additionally, 28 healthy controls scheduled for primary TJA during this period were matched for age and comorbidities with PJI cases and served as a control group (PA group) to evaluate baseline biomarker levels. Control participants met all inclusion criteria except for the criterion regarding revision surgery. In addition, the independent validation group included 22 patients with PJI and 28 patients with AF from June 2024 to January 2025. The enrollment flowchart for the patients is shown in **Figure 1**.

### Baseline data collection

Demographic and baseline clinical data were collected upon admission, including age, gender, height, weight, body mass index (BMI), comorbid conditions, American Society of Anesthesiologists (ASA) score, and affected joint type. Preoperative synovial fluid cultures were documented for PJI patients, with additional 3–6 samples of synovial fluid or peri-prosthetic tissue collected intraoperatively for microbial culture. The histological analysis or synovial fluid tests were performed.

Ultimately, this prospective study included 82 patients, with the baseline characteristics detailed in **Table 1**. The average age of the PJI patients in this group was 62.32 ± 11.25 years, with 16 men and 12 women. No significant demographic differences were observed among the three groups (all *P*>0.05).

**Table 1:** Demographics of patients included in this study.

Demographic variables	Total (N=82)	PJI (N=28)	AF (N=26)	PA (N=28)	*P value*
Age (year)	61.56±12.50	62.32±11.25	63.92±9.70	62.32±11.25	0.387
BMI (kg/m^2^)	25.93±7.13	26.20±8.15	25.74±5.12	25.94±8.32	0.747
Sex					0.076
Man	34 (41.5)	16 (57.1)	7 (26.9)	15 (53.6)	
Woman	48 (58.5)	12 (42.9)	19 (73.1)	13 (46.4)	
Joint					0.956
Hip	43 (52.4)	15 (53.6)	13 (50.0)	15 (53.6)	
Knee	39 (47.6)	13 (46.4)	13 (50.0)	13 (46.4)	
ASA score	2.17±0.43	2.20±0.40	2.18±0.51	2.14±0.39	0.198
CRP (mg/dL)	–	2.68±3.13	0.26±0.30	0.16±0.15	**<0.001**
ESR (mm/h)	–	39.61±25.96	14.38±13.55	6.42±3.16	**<0.001**
IL-6 (pg/mL)	–	20.62±16.72	3.36±2.65	3.54±7.66	**<0.001**

PJI, periprosthetic joint infection; AF, aseptic failure; PA, primary arthroplasty; CRP, C-reactive protein; ESR, erythrocyte sedimentation rate.

The values in bold indicate statistically significant differences.

### Sample collection and immune parameters analysis

Peripheral venous blood samples were collected from all eligible patients on the morning of the second hospital day, following an overnight fast. Samples were promptly processed within thirty minutes of collection at the laboratory. No patients were receiving antimicrobial therapy at the time of sample collection. The immune and inflammatory markers that were tested included: (1) traditional inflammatory markers: C-reactive protein (CRP), erythrocyte sedimentation rate (ESR), and interleukin-6 (IL-6); (2) lymphocyte subpopulations: percentages and absolute counts of NK cells, B cells, T cells, CD3+/CD4+ T cells, CD3+/CD8+ T cells, and the CD4/CD8 ratio; (3) serum immune-related proteins: complements C3 and C4, immunoglobulins (Ig) A, IgE, IgG, IgM, light chains κ and λ, β2-microglobulin, prealbumin, serum transferrin, ceruloplasmin, α1-acid glycoprotein, haptoglobin, β1-globulin, β2-globulin, albumin (ALB), α1-globulin, α2-globulin, and γ-globulin. All of the above tests were routinely available at our institution. Flow cytometry was used for lymphocyte subpopulation analysis (BD FACSLyric™ Flow Cytometer), and nephelometry immunoassay was used for immune-related protein detection (Siemens BN™ II System). In addition, haptoglobin levels were measured in the validation group patients to further support the current data.

### Data analyses

The quantitative data were expressed as mean ± standard deviation, while the categorical data were expressed as frequencies and percentages. The t-test and the Wilcoxon rank-sum test were used for between-group comparisons of normally and non-normally distributed variables, respectively; the ANOVA and the Kruskal-Wallis tests were applied for comparisons among multiple groups. The chi-square test was utilized for categorical comparisons. Pearson correlation analysis was employed to assess associations between immunological and serum inflammatory markers. Principal component analysis (PCA) was employed to reduce the dimensionality of the data set, thereby minimizing complexity while retaining essential variance, in order to evaluate global differences across groups. Receiver operating characteristic (ROC) curves were constructed to evaluate the predictive accuracy of various markers for PJI, and corresponding sensitivity, specificity and area under the curve (AUC) were calculated. The optimal cutoff value for each potential marker was determined using the Youden index. The combination of different immune-related markers and traditional blood markers was evaluated in testing the diagnostic efficacy of the combined indicators. The Delong test was used to compare the AUC of each diagnostic indicator. All statistical analyses were conducted using R software (version 3.8.1, R Development Core Team, Auckland, New Zealand), with a significance threshold set at *P* < 0.05.

## Results

### Lymphocyte subgroup

**Figure 2:**
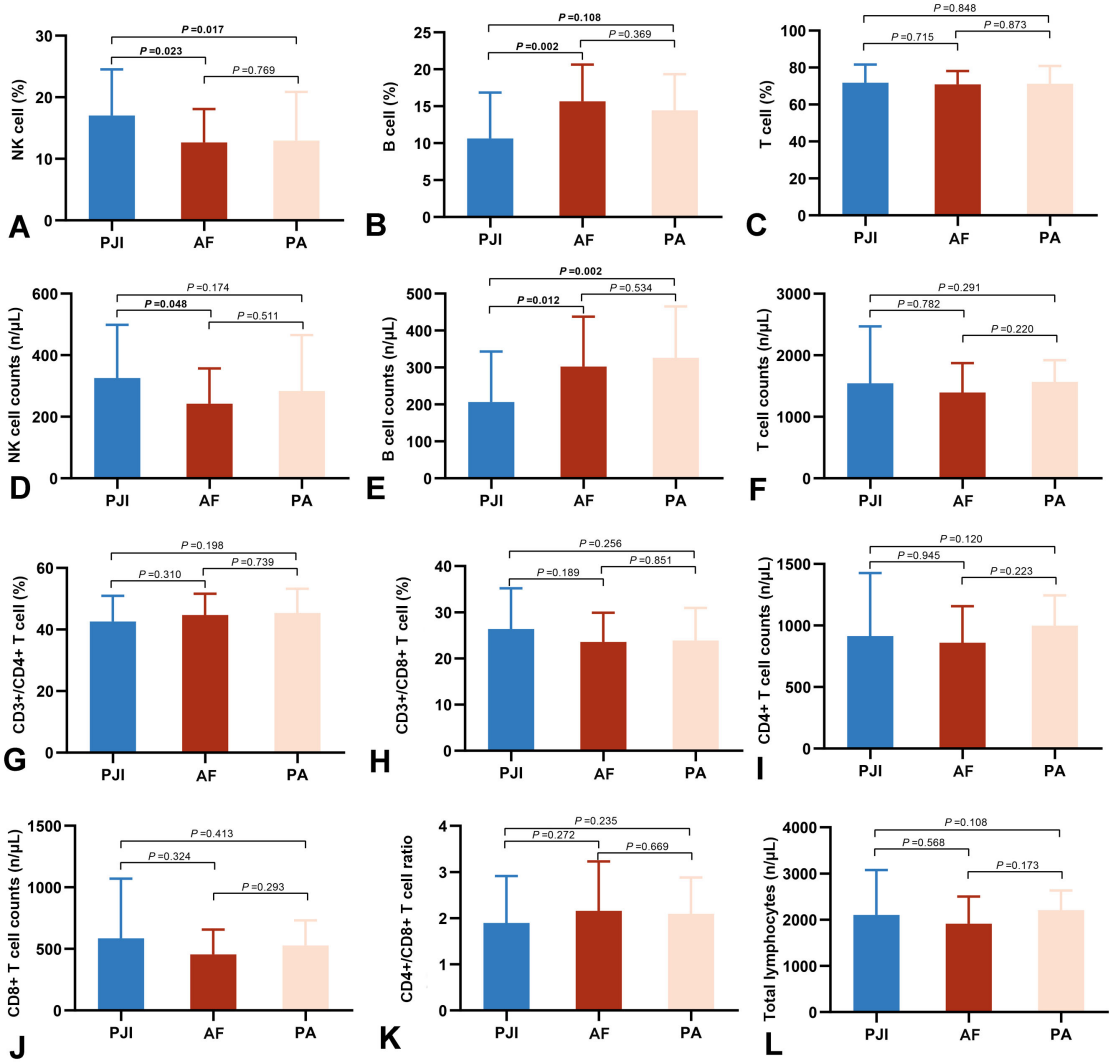
Comparison of peripheral lymphocyte subsets in the three groups, including percentages and absolute counts of NK cells **(A, D)**, B cells **(B, E)**, T cells **(C, F)**, CD3+/CD4+ T cells **(G)**, CD3+/CD8+Tcells **(H)**, CD4+ T cell counts **(I)**, CD8+ T cell counts **(J)**, CD4/CD8 ratio **(K)**, and total lymphocytes **(L)**. PJI, periprosthetic joint infection; AF, aseptic failure; PA, primary arthroplasty.

Peripheral blood lymphocyte levels are presented in **Figure 2** and **Supplementary Table 1**. The PJI group exhibited significantly higher NK cell proportion and absolute counts than the AF group (17.05 ± 7.49% vs. 12.67 ± 5.43%, *P* = 0.023; 325.60 ± 172.92 cells/μL vs. 242.23 ± 114.90 cells/μL, *P* = 0.017, respectively). Conversely, B cell proportion and absolute counts were lower in the PJI group compared to the AF group (10.63 ± 6.23% vs. 15.65 ± 4.98%, *P* = 0.002; 206.62 ± 136.58 cells/μL vs. 302.71 ± 135.08 cells/μL, *P* = 0.012, respectively). Similar trends were observed between the PJI and PA groups. However, we did not observe any changes in T cells and in the two sub-populations of CD4+ T cells and CD8+ T cells. No any lymphocyte indicators differed significantly between the AF and PA groups.

### Immune-related protein spectrum

**Figure 3:**
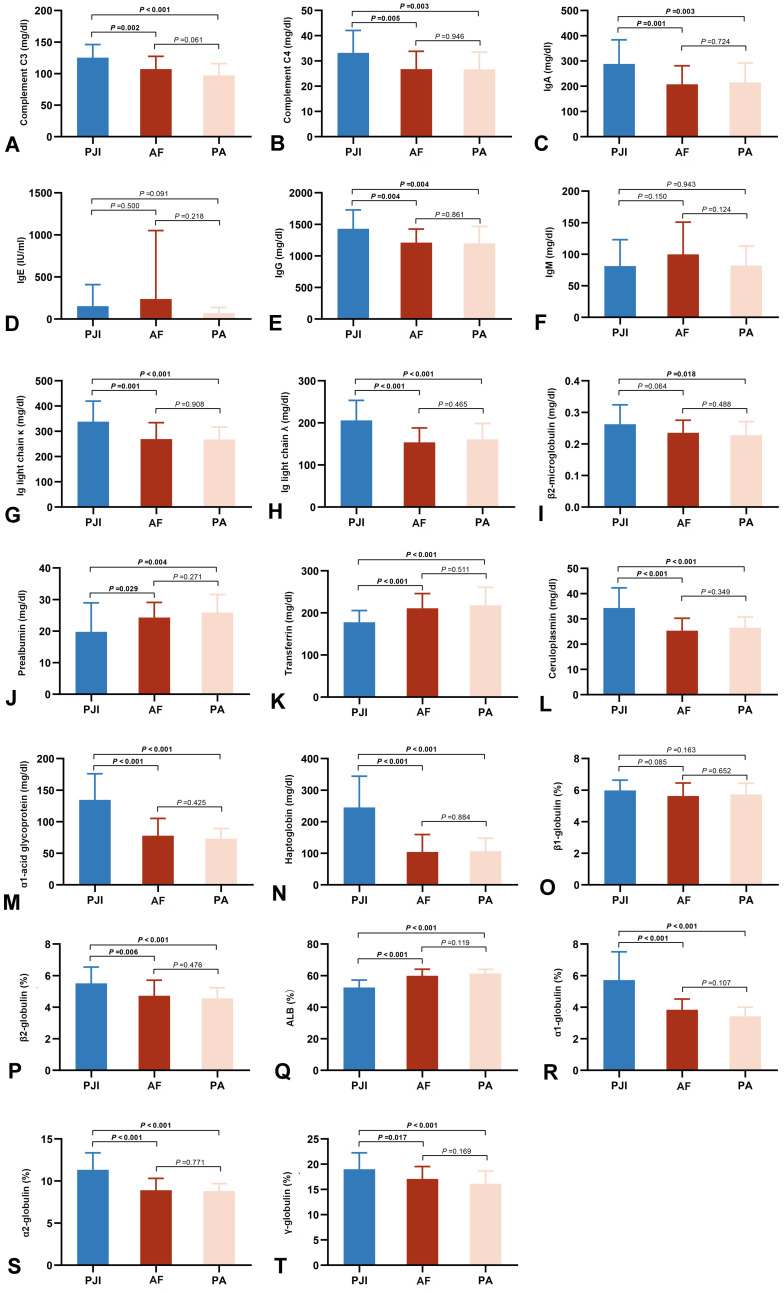
Comparison of peripheral immune-related proteins in the three groups, including complements C3 **(A)** and C4 **(B)**, Ig A **(C)**, IgE **(D)**, IgG **(E)**, IgM **(F)**, light chains κ **(G)** and λ **(H)**, β2-microglobulin **(I)**, prealbumin **(J)**, serum transferrin **(K)**, ceruloplasmin **(L)**, α1-acid glycoprotein **(M)**, haptoglobin **(N)**, β1-globulin **(O)**, β2-globulin **(P)**, albumin **(Q)**, α1-globulin **(R)**, α2-globulin **(S)**, and γ-globulin **(T)**. PJI, periprosthetic joint infection; AF, aseptic failure; PA, primary arthroplasty.

The majority of immune-related proteins exhibited significant differences between the PJI and AF groups(**Figure 3** and **Supplementary Table 2**). Immunoglobulins and the complement system were systemically activated in PJI patients. Among them, the increases in α1-acid glycoprotein (134.58 ± 41.30 mg/dl vs. 77.80 ± 27.31 mg/dl, *P* < 0.001, **Figure 3M**) and haptoglobin (245.08 ± 99.00 mg/dl vs. 108.22 ± 52.37 mg/dl, *P* < 0.001, **Figure 3N**) were the most pronounced. Similar disparities were noted between the PJI and PA groups.

**Figure 4:**
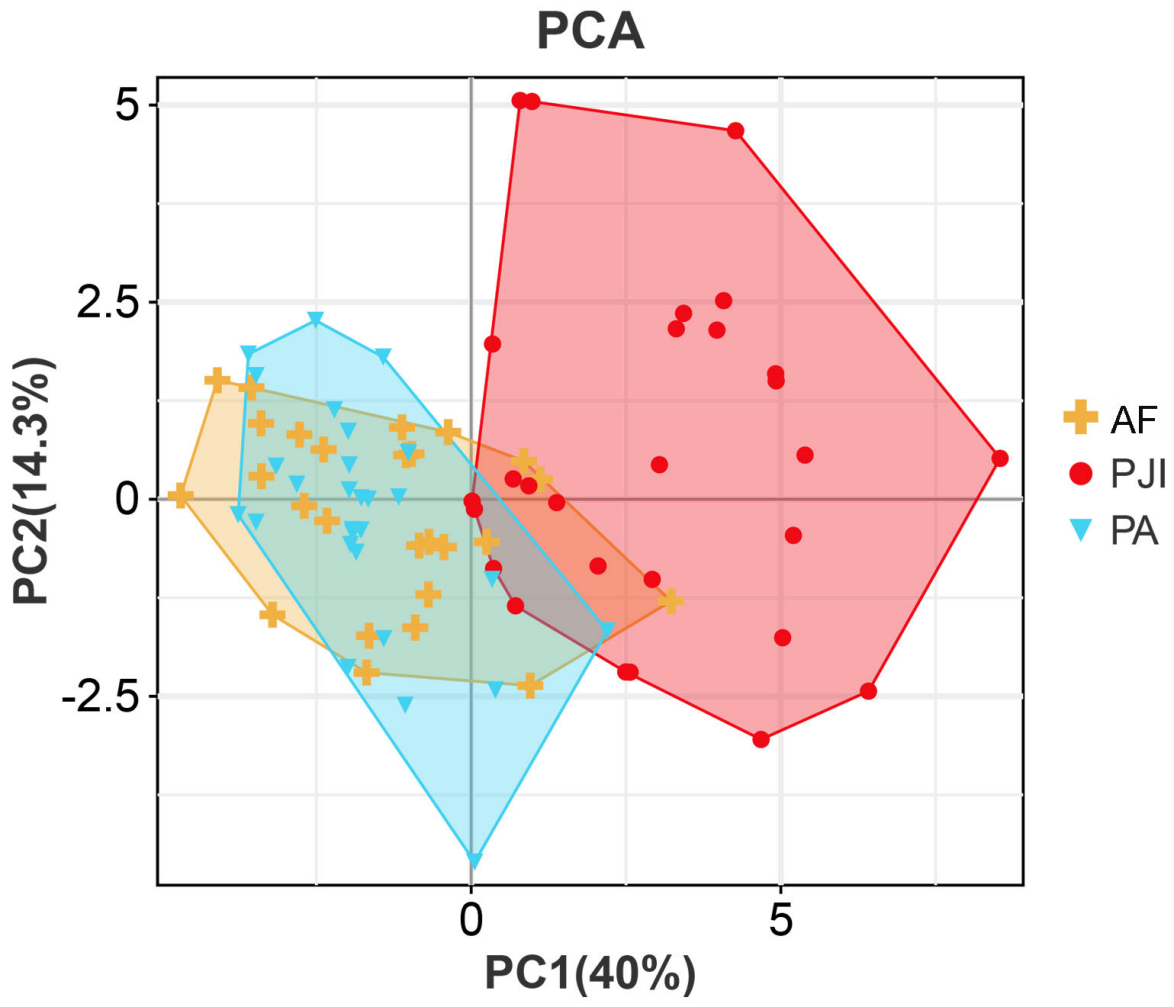
Principal component analysis of immune parameters. PJI, periprosthetic joint infection; AF, aseptic failure; PA, primary arthroplasty.

The PCA results demonstrated that the differential immune indicators could effectively distinguish systemic immune profiles. A clear clustering pattern showed segregation of the PJI group from the AF and PA groups (**Figure 4**). The first two principal components (PC1 and PC2) explained over 50% of the total variance, with PC1 primarily driving the separation. This indicates that significant systemic immunologic changes are characteristic of PJI, while such changes are minimal or absent between patients with aseptic failure and primary arthroplasty. Correlations were observed between the majority of immune markers and inflammatory markers, including CRP, ESR, and IL-6 (**Table 2**). Haptoglobin and α1-acid glycoprotein showed relatively strong correlations with inflammatory indicators, with correlation coefficients around 0.7.

**Table 2:** Correlation analysis of peripheral immune parameters and inflammatory indicators.

Immune-related markers	CRP (mg/dL)	ESR (mm/h)	IL-6 (pg/mL)
NK cell (%)	**r = 0.264** ***P* = 0.016**	**r = 0.246** ***P* = 0.026**	**r = 0.226** ***P* = 0.041**
Absolute NK cell counts (n/μL)	r = 0.009 *P* = 0.938	r = 0.116 *P* = 0.301	r = 0.061 *P* = 0.588
B cell (%)	r = -0.161 *P* = 0.149	**r = -0.272** ***P* = 0.013**	**r = -0.331** ***P* = 0.002**
Absolute B cell counts (n/μL)	**r = -0.306** ***P* = 0.005**	**r = -0.261** ***P* = 0.018**	**r = -0.344** ***P* = 0.002**
Complement C3 (mg/dl)	**r = 0.373** ***P* = 0.001**	**r = 0.531** ***P* < 0.001**	**r = 0.274** ***P* = 0.013**
Complement C4 (mg/dl)	r = 0.198 *P* = 0.075	**r = 0.397** ***P* < 0.001**	r = 0.199 *P* = 0.073
IgA (mg/dl)	**r = 0.317** ***P* = 0.004**	**r = 0.351** ***P* = 0.001**	**r = 0.356** ***P* = 0.001**
IgG (mg/dl)	**r = 0.403** ***P* < 0.001**	**r = 0.463** ***P* < 0.001**	**r = 0.235** ***P* = 0.034**
Ig light chain κ (mg/dl)	**r = 0.531** ***P* = < 0.001**	**r = 0.538** ***P* < 0.001**	**r = 0.334** ***P* = 0.002**
Ig light chain λ (mg/dl)	**r = 0.428** ***P* < 0.001**	**r = 0.466** ***P* < 0.001**	**r = 0.316** ***P* = 0.004**
Prealbumin (mg/dl)	**r = -0.407** ***P* < 0.001**	**r = -0.466** ***P* < 0.001**	**r = -0.459** ***P* < 0.001**
Transferrin (mg/dl)	**r = -0.305** ***P* = 0.005**	**r = -0.435** ***P* < 0.001**	**r = -0.234** ***P* = 0.034**
Ceruloplasmin (mg/dl)	**r = 0.541** ***P* < 0.001**	**r = 0.728** ***P* < 0.001**	**r = 0.466** ***P* < 0.001**
α1-acid glycoprotein (mg/dl)	**r = 0.758** ***P* < 0.001**	**r = 0.750** ***P* < 0.001**	**r = 0.706** ***P* < 0.001**
Haptoglobin (mg/dl)	**r = 0.707** ***P* < 0.001**	**r = 0.661** ***P* < 0.001**	**r = 0.674** ***P* < 0.001**
β2-globulin (%)	**r = 0.372** ***P* = 0.001**	**r = 0.416** ***P* < 0.001**	**r = 0.456** ***P* < 0.001**
ALB (%)	**r = -0.666** ***P* < 0.001**	**r = -0.757** ***P* < 0.001**	**r = -0.647** ***P* < 0.001**
α1-globulin (%)	**r = 0.549** ***P* < 0.001**	**r = 0.584** ***P* < 0.001**	**r = 0.568** ***P* < 0.001**
α2-globulin (%)	**r = 0.531** ***P* < 0.001**	**r = 0.621** ***P* < 0.001**	**r = 0.612** ***P* < 0.001**
γ-globulin (%)	**r = 0.442** ***P* < 0.001**	**r = 0.531** ***P* < 0.001**	**r = 0.307** ***P* = 0.005**

CRP, C-reactive protein; ESR, erythrocyte sedimentation rate.

The values in bold indicate statistically significant differences.

### Diagnostic potential of immune-related parameters

**Figure 5:**
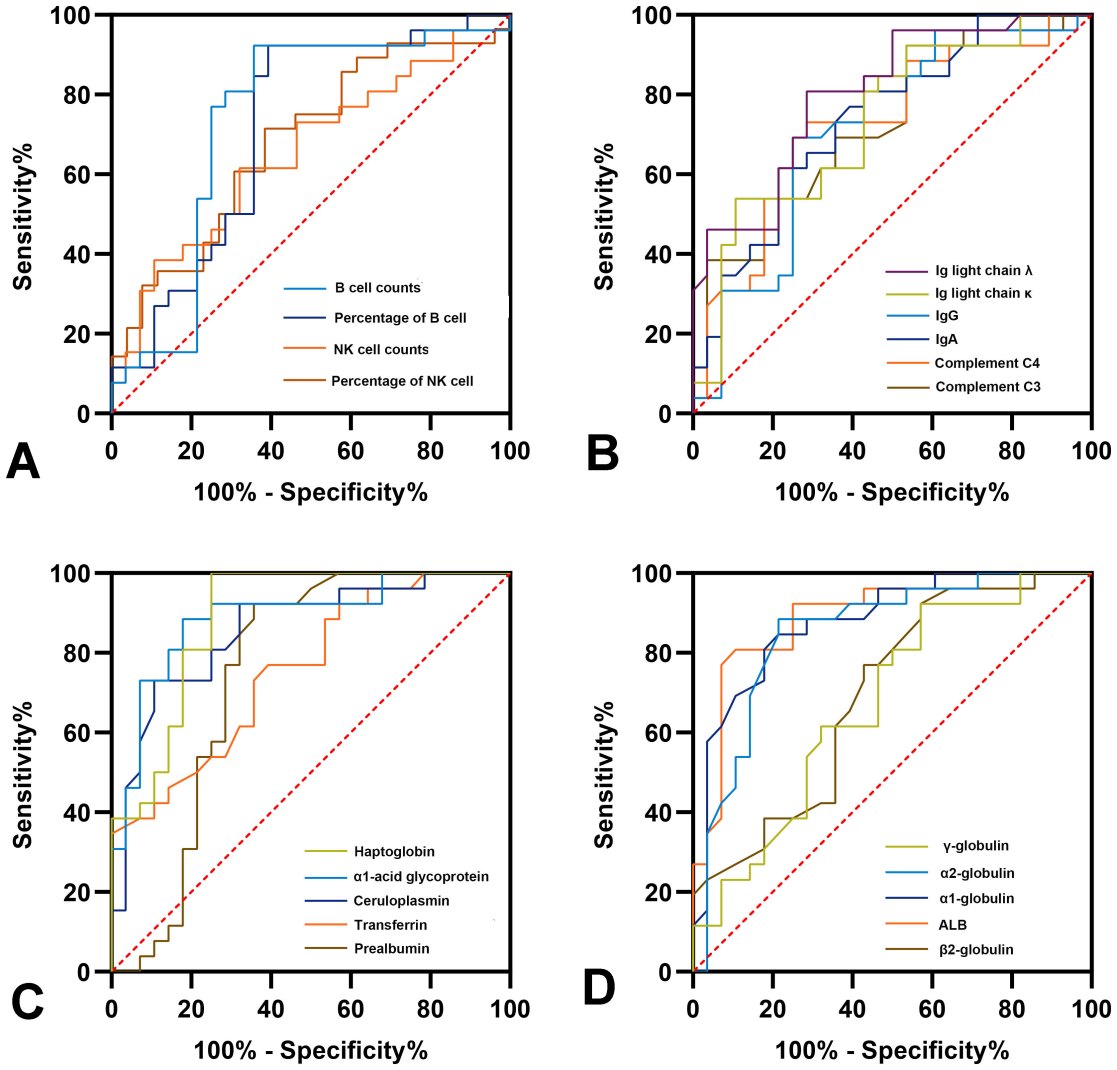
Receiver operator characteristic curves of immune parameters for diagnosis of PJI.

The potential of the different immune indicators as novel diagnostic markers for PJI was evaluated using ROC analysis, with the results detailed in **Figure 5** and **Supplementary Table 3**. Among the lymphocyte subgroups, absolute B cell counts exhibited the best diagnostic performance (AUC = 0.735, 95%CI: 0.590-0.880). Among the immune-related proteins, haptoglobin had the best diagnostic accuracy (AUC = 0.890, 95%CI: 0.803-0.978), which is comparable to CRP (AUC = 0.900, *P*>0.05) while better than ESR (AUC = 0.837, *P* = 0.017). Notably, the haptoglobin achieved a sensitivity of 100% and a specificity of 75% at a cutoff of 186.5 mg/dL.

The same trend was observed in the validation group. Haptoglobin levels were significantly higher in patients with PJI (227.08 ± 92.00 mg/dl vs. 119.72 ± 78.97 mg/dl, P < 0.001) and had a diagnostic efficacy comparable to CRP (AUC: 0.856 vs. 0.880).

**Figure 6:**
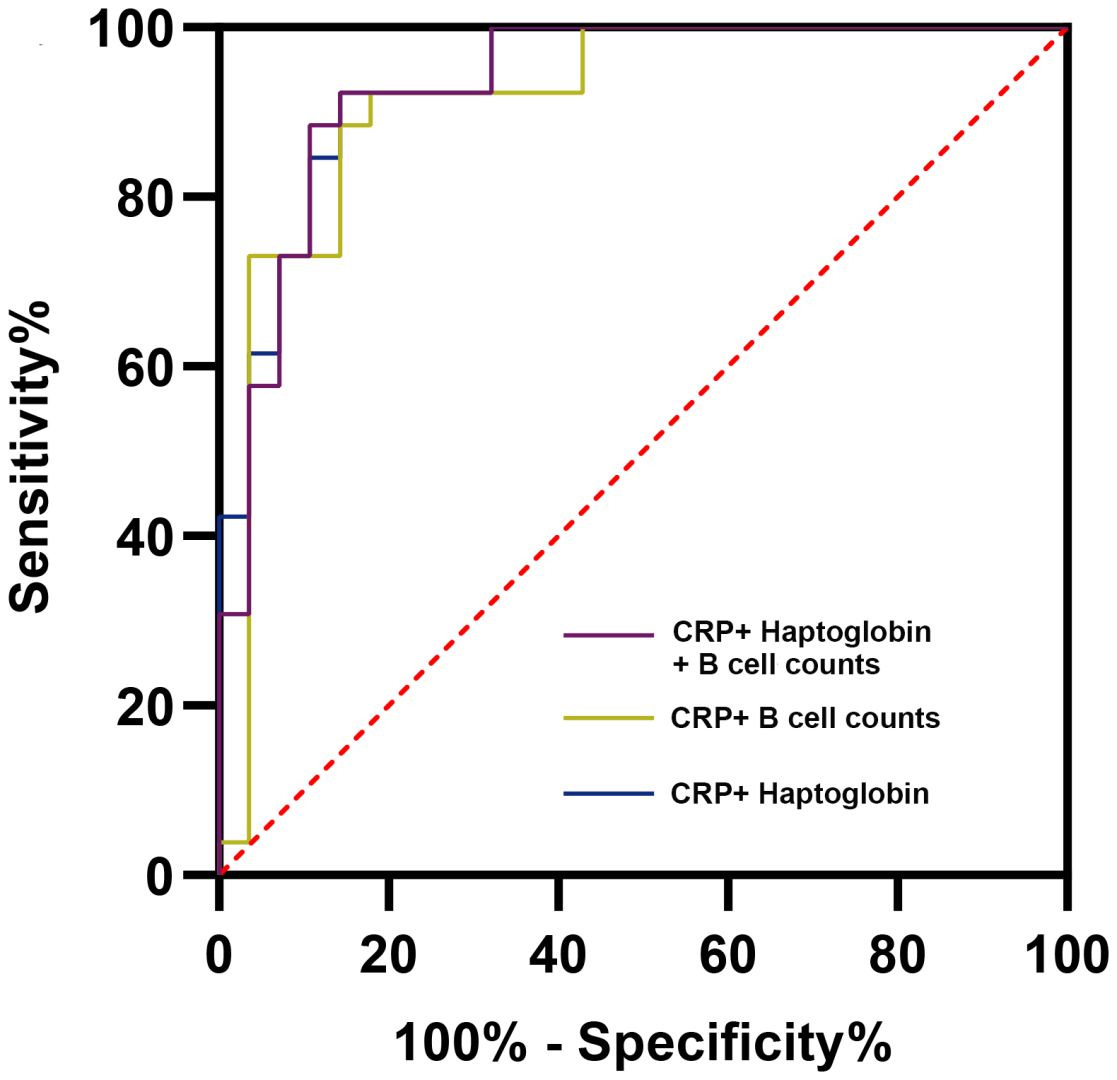
Receiver operator characteristic curves of representative combined parameters for the diagnosis of periprosthetic joint infection. CRP, C-reactive protein.

We evaluated various combinations and found that the combination of CRP and haptoglobin had the highest diagnostic efficacy (AUC = 0.937, 95% CI: 0.876-0.998), which was significantly better than the combination of CRP and ESR (AUC = 0.886, *P* = 0.025). In addition, CRP+B cell absolute counts demonstrated the best diagnostic ability in combinations that included lymphocyte subsets (AUC = 0.914, 95% CI: 0.831-0.996). These results indicated that the immune markers could further enhance the diagnostic ability of the serum indicators. Combining any three parameters did not further improve diagnostic accuracy (AUC for haptoglobin + CRP + B cell absolute counts was highest at 0.933, 95% CI: 0.866-0.999, **Figure 6**).

## Discussion

The systemic immune responses that are elicited by PJI remain poorly understood. In light of the intricate interrelationship between implant materials, infection, and the immune system, it is imperative to enhance our comprehension of the systemic immune profile in patients with PJI. This study is, to the best of our knowledge, the first to provide a systemic profile of peripheral blood lymphocyte subsets and immune-related proteins in PJI, and to comprehensively compare them with AF or PA groups. The present study revealed significant alterations in the lymphocyte profiles of PJI patients, characterized by decreases in B lymphocyte, and increases in NK lymphocyte. Moreover, the majority of immune-related protein levels in PJI patients demonstrated notable alterations. These immune parameters may offer supplementary insights into the diagnosis of PJI, especially the haptoglobin with comparable diagnostic accuracy of CRP. More significantly, the combination of haptoglobin and CRP can achieve superior diagnostic efficacy. However, the levels of immune-related indicators in AF patients were found to be comparable to those in the PA group.

This study is the first to document a paradoxical decrease in peripheral B cells with an increase in NK cells among patients with PJI. In the context of infectious diseases, lymphocytes play a pivotal role in the immune response to sepsis, and their numbers serve as a direct reflection of a patient’s immune status (15, 18). NK cells are integral components of innate immunity and are capable of killing target cells in an unactivated state during infectious diseases. However, the evidence regarding NK cell behavior in infection is contradictory. For example, a prospective study conducted by David Andaluz-Ojeda revealed that in intensive care units, sepsis survivors exhibited lower NK cell counts and percentage compared to non-survivors (19). Previous studies demonstrated that higher NK cell counts in sepsis was associated with poorer outcomes (20), whereas other clinical reports indicated that NK cells significantly increase during the early stages of sepsis (21).

B cells can be activated and increased by antigen stimulation during the initial stages of an infection. Peng et al. (22) observed elevated percentages of B cells in sepsis patients when compared to healthy controls. However, B cells were found to drastically decrease in patients with septic shock. Furthermore, impairments in B cell activation have been observed in the early stage of invasive pneumococcal infection (23). A reduction in naive B cells (CD^19+^CD^27–^), and an increase in immature B cells (CD19^+^CD5^+^CD27^–^CD21^–/low^) have been documented in elderly patients with sepsis (24). These findings indicated that B cells may enter an “exhausted” state during severe infections. We have preliminarily observed a reduction in peripheral B cells in patients with chronic PJI. We speculate that this may primarily result from persistent antigen stimulation derived from biofilms, leading to B cell exhaustion. Additionally, B cells may be recruited from circulation to the local infected joint tissue via chemokines. The above conjecture is preliminary, and further investigation into this phenomenon is warranted in the future. The profiling of peripheral lymphocyte subgroups in PJI patients could facilitate the development of new personalized diagnostic and treatment strategies by enhancing our understanding of the dynamic immune responses involved.

Activation of the complement system, a vital immune mechanism against infection, entails the engagement of multiple pathways that collectively activate C3 convertase (25). In our study, we observed systemic activation of the complement system; however, its accuracy as a diagnostic marker was insufficient, with an AUC of 0.725. Fröschen et al. (26) have measured complement factors in synovial fluid, suggesting that all complement pathways may be activated in PJI. However, they also observed that the diagnostic utility of individual complement factor was constrained. As demonstrated by the findings of Meinshausen et al., the terminal complement pathway was activated in infection following shoulder arthroplasty, whereas it remained normal in aseptic revisions (27). Another study, comprising 98 revision surgery patients, employed immunohistochemical staining of periprosthetic tissues to identify complement C9 as a potential diagnostic marker for PJI (sensitivity = 89%, specificity = 75%) (28). Besides, no significant differences in tissue C9 staining were observed between different pathogens, suggesting that it could serve as a new marker unaffected by confounding factors such as infection stage or pathogen type.

An inflammatory response is initiated when infection or tissue damage occurs, resulting in the release of pro-inflammatory cytokines and a subsequent systemic immune response. In such scenarios, the liver synthesizes haptoglobin as an acute-phase protein (APP) (29). Haptoglobin could bind to free hemoglobin in the periphery, thereby reducing its toxicity and depriving bacteria of the iron necessary for their metabolism (30). Therefore, haptoglobin plays a pivotal role in antibacterial responses and mediates a multitude of immune regulatory reactions, including inhibiting the oxidative activity of hemoglobin, reducing free radical production, and regulating the release of inflammatory factors. Remy et al. have found that infusion of haptoglobin could improve shock, lung injury, and survival in canine pneumonia (31). Additionally, it has the capacity to bind at two distinct sites on neutrophils, thereby inhibiting calcium influx and subsequent reactive oxygen production (32). This study was preliminary, but since then there have been few studies on the association between haptoglobin and neutrophils. Theilgaard Monch et al. subsequently demonstrated it could be synthesized and stored during granulocyte differentiation and released in response to neutrophil activation, thereby increasing its concentration in the periphery by severalfold following infection or tissue damage (29). Therefore, the potential association between haptoglobin and neutrophils should be further explored in the future. Haptoglobin has been demonstrated to be involved in the pathogenesis of a number of diseases, including those of the cardiovascular, neurological, and inflammatory systems (33). Chavez-Bueno et al. (34) have revealed the elevated level of haptoglobin in cases of neonatal bacteremia. Additionally, elevated cord blood haptoglobin level could serve as a predictor for neonatal sepsis (35). Moreover, elevated plasma haptoglobin level has been demonstrated to be associated with a reduced risk of in-hospital mortality, and this association is independent of confounding factors. The present study showed that the haptoglobin level of PJI patients was approximately 2.5 times higher than that of the control group. In addition, it has an excellent ability to identify PJI patients with a sensitivity of 100%. These findings suggested that haptoglobin can be used as an excellent serum screening marker. When used together with CRP for the diagnosis of PJI, they achieved an AUC of 0.937. These results could be further validated in studies with larger sample sizes.

Moreover, we found that another APP, α1-acid glycoprotein, was about twice as concentrated in PJI patients compared to controls and also exhibited good diagnostic value (AUC = 0.889). Mestriner et al. (36) has found that α1-acid glycoprotein can inhibit neutrophil migration to infection sites through a nitric oxide-dependent process and is involved in the pathogenesis of human sepsis. Importantly, some reports on animal models of bone infection suggested that α1-acid glycoprotein may be a more characteristic serum marker of infection than CRP (37–39). It was noteworthy that the AUC of ceruloplasmin for diagnosing PJI also reached 0.859. These findings indicated that further investigation into the roles of various immune-related APPs in the pathogenesis of PJI and their potential as new immune markers was warranted.

The present study has several limitations. Due to sample size limitations, we did not perform subgroup analyses of immune responses induced by each pathogen. Besides, the range of peripheral immune-related indicators was extensive, and the immune markers included in this study were determined based on previous literature, laboratory availability, and PJI expert opinion. Future investigation of additional immune-related markers may provide additional valuable insights. In addition, the immune system is highly dynamic, and data from a single time point may not accurately reflect the entire disease process. Longer-term follow-up studies may provide more valuable information.

## Conclusions

Significant changes in peripheral lymphocyte subsets were observed in PJI patients, characterized by a decrease in B lymphocytes and an increase in NK lymphocytes. In addition, most peripheral immune-related protein levels showed significant changes in PJI patients. However, systemic immune markers in AF patients were comparable to those in patients undergoing primary arthroplasty. Several systemic immune markers showed promising diagnostic ability for PJI, and the diagnostic efficacy of haptoglobin was comparable to that of CRP. Combining specific immune-related markers with traditional inflammatory biomarkers could further improve the diagnostic efficacy of serum parameters.

## Data Availability

The original contributions presented in the study are included in the article/**supplementary files**, further inquiries can be directed to the corresponding author/s.

## References

[1] Singh JA Yu S Chen L Cleveland JD . Rates of total joint replacement in the United States: future projections to 2020–2040 using the national inpatient sample. J Rheumatol. (2019) 46:1134–40. doi: 10.3899/jrheum.170990, PMID: 30988126

[2] Kurtz S Ong K Lau E Mowat F Halpern M . Projections of primary and revision hip and knee arthroplasty in the United States from 2005 to 2030. J Bone Joint Surg Am. (2007) 89:780–5. doi: 10.2106/00004623-200704000-00012, PMID: 17403800

[3] Quinlan ND Werner BC Brown TE Browne JA . Risk of prosthetic joint infection increases following early aseptic revision surgery of total hip and knee arthroplasty. J Arthropl. (2020) 35:3661–7. doi: 10.1016/j.arth.2020.06.089, PMID: 32712119

[4] Xu C Goswami K Li WT Tan TL Yayac M Wang SH . Is treatment of periprosthetic joint infection improving over time? J Arthropl. (2020) 35:1696–702 e1. doi: 10.1016/j.arth.2020.01.080, PMID: 32192834

[5] Li C Ojeda-Thies C Renz N Margaryan D Perka C Trampuz A . The global state of clinical research and trends in periprosthetic joint infection: A bibliometric analysis. Int J Infect Dis. (2020) 96:696–709. doi: 10.1016/j.ijid.2020.05.014, PMID: 32434084

[6] Li Z Maimaiti Z Li ZY Fu J Hao LB Xu C . Moderate-to-severe malnutrition identified by the controlling nutritional status (CONUT) score is significantly associated with treatment failure of periprosthetic joint infection. Nutrients. (2022) 14:4433. doi: 10.3390/nu14204433, PMID: 36297116 PMC9607573

[7] Li Z Maimaiti Z Fu J Li ZY Hao LB Xu C . The superiority of immune-inflammation summary index for diagnosing periprosthetic joint infection. Int Immunopharmacol. (2023) 118:110073. doi: 10.1016/j.intimp.2023.110073, PMID: 36989888

[8] Li Z Li ZY Maimaiti Z Yang F Fu J Hao LB . Identification of immune infiltration and immune-related biomarkers of periprosthetic joint infection. Heliyon. (2024) 10:e26062. doi: 10.1016/j.heliyon.2024.e26062, PMID: 38370241 PMC10867348

[9] Korn MF Stein RR Dolf A Shakeri F Buness A Hilgers C . High-dimensional analysis of immune cell composition predicts periprosthetic joint infections and dissects its pathophysiology. Biomedicines. (2020) 8:358. doi: 10.3390/biomedicines8090358, PMID: 32957521 PMC7554968

[10] Jubel JM Randau TM Becker-Gotot J Scheidt S Wimmer MD Kohlhof H . sCD28, sCD80, sCTLA-4, and sBTLA Are Promising Markers in Diagnostic and Therapeutic Approaches for Aseptic Loosening and Periprosthetic Joint Infection. Front Immunol. (2021) 12:687065. doi: 10.3389/fimmu.2021.687065, PMID: 34421900 PMC8377391

[11] Fisher CR Krull JE Bhagwate A Masters T Greenwood-Quaintance KE Abdel MP . Sonicate fluid cellularity predicted by transcriptomic deconvolution differentiates infectious from non-infectious arthroplasty failure. J Bone Joint Surg Am. (2023) 105:63–73. doi: 10.2106/JBJS.22.00605, PMID: 36574631 PMC10137834

[12] Vantucci CE Ahn H Fulton T Schenker ML Pradhan P Wood LB . Development of systemic immune dysregulation in a rat trauma model of biomaterial-associated infection. Biomaterials. (2021) 264:120405. doi: 10.1016/j.biomaterials.2020.120405, PMID: 33069135 PMC8117743

[13] Sadtler K Estrellas K Allen BW Wolf MT Fan H Tam AJ . Developing a pro-regenerative biomaterial scaffold microenvironment requires T helper 2 cells. Science. (2016) 352:366–70. doi: 10.1126/science.aad9272, PMID: 27081073 PMC4866509

[14] Partridge DG Winnard C Townsend R Cooper R Stockley I . Joint aspiration, including culture of reaspirated saline after a ‘dry tap’, is sensitive and specific for the diagnosis of hip and knee prosthetic joint infection. Bone Joint J. (2018) 100-B:749–54. doi: 10.1302/0301-620X.100B6.BJJ-2017-0970.R2, PMID: 29855250

[15] Zhao J Dai RS Chen YZ Zhuang YG . Prognostic significance of lymphocyte subpopulations for ICU-acquired infections in patients with sepsis: a retrospective study. J Hosp Infect. (2023) 140:40–5. doi: 10.1016/j.jhin.2023.05.022, PMID: 37399906

[16] Boomer JS Shuherk-Shaffer J Hotchkiss RS Green JM . A prospective analysis of lymphocyte phenotype and function over the course of acute sepsis. Crit Care. (2012) 16:R112. doi: 10.1186/cc11404, PMID: 22742734 PMC3580670

[17] Parvizi J Gehrke T . International Consensus Group on Periprosthetic Joint I. Definition of periprosthetic joint infection. J Arthropl. (2014) 29:1331. doi: 10.1016/j.arth.2014.03.009, PMID: 24768547

[18] Esposito S De Simone G Boccia G De Caro F Pagliano P . Sepsis and septic shock: New definitions, new diagnostic and therapeutic approaches. J Glob Antimicrob Resist. (2017) 10:204–12. doi: 10.1016/j.jgar.2017.06.013, PMID: 28743646

[19] Andaluz-Ojeda D Iglesias V Bobillo F Almansa R Rico L Gandia F . Early natural killer cell counts in blood predict mortality in severe sepsis. Crit Care. (2011) 15:R243. doi: 10.1186/cc10501, PMID: 22018048 PMC3334794

[20] Guo Y Patil NK Luan L Bohannon JK Sherwood ER . The biology of natural killer cells during sepsis. Immunology. (2018) 153:190–202. doi: 10.1111/imm.12854, PMID: 29064085 PMC5765373

[21] Giamarellos-Bourboulis EJ Tsaganos T Spyridaki E Mouktaroudi M Plachouras D Vaki I . Early changes of CD4-positive lymphocytes and NK cells in patients with severe Gram-negative sepsis. Crit Care. (2006) 10:R166. doi: 10.1186/cc5111, PMID: 17129388 PMC1794479

[22] Peng Y Wang X Yin S Wang M . A new indicator: The diagnostic value of CD8+T/B lymphocyte ratio in sepsis progression. Int J Immunopathol Pharmacol. (2022) 36:3946320221123164. doi: 10.1177/03946320221123164, PMID: 36036157 PMC9421217

[23] Monserrat J de Pablo R Diaz-Martin D Rodriguez-Zapata M de la Hera A Prieto A . Early alterations of B cells in patients with septic shock. Crit Care. (2013) 17:R105. doi: 10.1186/cc12750, PMID: 23721745 PMC4056890

[24] Suzuki K Inoue S Kametani Y Komori Y Chiba S Sato T . Reduced immunocompetent B cells and increased secondary infection in elderly patients with severe sepsis. Shock. (2016) 46:270–8. doi: 10.1097/SHK.0000000000000619, PMID: 27172158

[25] Gros P Milder FJ Janssen BJ . Complement driven by conformational changes. Nat Rev Immunol. (2008) 8:48–58. doi: 10.1038/nri2231, PMID: 18064050

[26] Froschen FS Schell S Wimmer MD Hischebeth GTR Kohlhof H Gravius S . Synovial complement factors in patients with periprosthetic joint infection after undergoing revision arthroplasty of the hip or knee joint. Diagn (Basel). (2021) 11:434. doi: 10.3390/diagnostics11030434, PMID: 33806309 PMC8002017

[27] Meinshausen AK Martens N Berth A Farber J Awiszus F Macor P . The terminal complement pathway is activated in septic but not in aseptic shoulder revision arthroplasties. J Shoulder Elbow Surg. (2018) 27:1837–44. doi: 10.1016/j.jse.2018.06.037, PMID: 30139682

[28] Meinshausen AK Farber J Illiger S Macor P Lohmann CH Bertrand J . C9 immunostaining as a tissue biomarker for periprosthetic joint infection diagnosis. Front Immunol. (2023) 14:1112188. doi: 10.3389/fimmu.2023.1112188, PMID: 36895567 PMC9989178

[29] Theilgaard-Monch K Jacobsen LC Nielsen MJ Rasmussen T Udby L Gharib M . Haptoglobin is synthesized during granulocyte differentiation, stored in specific granules, and released by neutrophils in response to activation. Blood. (2006) 108:353–61. doi: 10.1182/blood-2005-09-3890, PMID: 16543473

[30] Lim SK Ferraro B Moore K Halliwell B . Role of haptoglobin in free hemoglobin metabolism. Redox Rep. (2001) 6:219–27. doi: 10.1179/135100001101536364, PMID: 11642712

[31] Remy KE Cortes-Puch I Solomon SB Sun J Pockros BM Feng J . Haptoglobin improves shock, lung injury, and survival in canine pneumonia. JCI Insight. (2018) 3:e123013. doi: 10.1172/jci.insight.123013, PMID: 30232287 PMC6237235

[32] Oh SK Pavlotsky N Tauber AI . Specific binding of haptoglobin to human neutrophils and its functional consequences. J Leukoc Biol. (1990) 47:142–8. doi: 10.1002/jlb.47.2.142, PMID: 2303749

[33] MacKellar M Vigerust DJ . Role of haptoglobin in health and disease: A focus on diabetes. Clin Diabetes. (2016) 34:148–57. doi: 10.2337/diaclin.34.3.148, PMID: 27621532 PMC5019011

[34] Chavez-Bueno S Beasley JA Goldbeck JM Bright BC Morton DJ Whitby PW . ‘Haptoglobin concentrations in preterm and term newborns’. J Perinatol. (2011) 31:500–3. doi: 10.1038/jp.2010.197, PMID: 21252963

[35] Mithal LB Palac HL Yogev R Ernst LM Mestan KK . Cord blood acute phase reactants predict early onset neonatal sepsis in preterm infants. PloS One. (2017) 12:e0168677. doi: 10.1371/journal.pone.0168677, PMID: 28045978 PMC5207723

[36] Mestriner FL Spiller F Laure HJ Souto FO Tavares-Murta BM Rosa JC . Acute-phase protein alpha-1-acid glycoprotein mediates neutrophil migration failure in sepsis by a nitric oxide-dependent mechanism. Proc Natl Acad Sci U S A. (2007) 104:19595–600. doi: 10.1073/pnas.0709681104, PMID: 18048324 PMC2148334

[37] Soe NH Jensen NV Nurnberg BM Jensen AL Koch J Poulsen SS . A novel knee prosthesis model of implant-related osteomyelitis in rats. Acta Orthop. (2013) 84:92–7. doi: 10.3109/17453674.2013.773121, PMID: 23409845 PMC3584611

[38] Wei J Wen Y Tong K Wang H Chen L . Local application of vancomycin in one-stage revision of prosthetic joint infection caused by methicillin-resistant staphylococcus aureus. Antimicrob Agents Chemother. (2021) 65:e0030321. doi: 10.1128/AAC.00303-21, PMID: 34181479 PMC8370217

[39] Cray C Zaias J Altman NH . Acute phase response in animals: a review. Comp Med. (2009) 59:517–26. PMC2798837 20034426

