# Supplementary material for: Significant alterations in peripheral lymphocyte subsets and immune-related protein profiles in patients with periprosthetic joint infection
Source: Front Immunol. 2025 Nov 19;16:1648150. doi: 10.3389/fimmu.2025.1648150 (PMC12672869; doi:10.3389/fimmu.2025.1648150)
Supplement: Supplementary file 1 [file Table1.docx]

**Supplementary Table 1. Results of peripheral blood lymphocyte subsets in each group.** PJI, periprosthetic joint infection; AF, aseptic failure; PA, primary arthroplasty

|  | **PJI (N=28)** | **AF (N=26)** | **PA (N=28)** |
| --- | --- | --- | --- |
| **NK cell (%)** | 17.05±7.49 | 12.67±5.43 | 12.97±7.89 |
| **Absolute NK cell counts (n/μL)** | 325.60±172.92 | 242.23±114.90 | 283.95±181.26 |
| **B cell (%)** | 10.63±6.23 | 15.65±4.98 | 13.82±5.76 |
| **Absolute B cell counts (n/μL)** | 206.62±136.58 | 302.71±135.08 | 277.88±145.08 |
| **T cell (%)** | 71.76±9.85 | 70.89±7.25 | 71.32±8.89 |
| **Absolute T cell counts (n/μL)** | 1543.50±926.24 | 1394.32±477.60 | 1504.13±635.27 |
| **CD3+/CD4+T (%)** | 42.60±8.32 | 44.74±6.91 | 45.42±7.85 |
| **CD3+/CD8+T (%)** | 26.35±8.82 | 23.56±6.33 | 23.90±7.05 |
| **CD4/CD8 ratio** | 1.90±1.02 | 2.16±1.07 | 2.05±0.96 |
| **Absolute CD4+ T cell counts (n/μL)** | 914.93±511.11 | 860.31±295.68 | 926.38±370.87 |
| **Absolute CD8+ T cell counts (n/μL)** | 586.27±483.42 | 454.55±202.06 | 527.19±202.52 |
| **Total lymphocytes (n/μL)** | 2103.66±972.31 | 1915.00±588.32 | 2205.90±428.81 |

**Supplementary Table 2. Results of peripheral blood immune-related proteins in each group.** PJI, periprosthetic joint infection; AF, aseptic failure; PA, primary arthroplasty

|  | **PJI (n=28)** | **AF (n=26)** | **PA (n=28)** |
| --- | --- | --- | --- |
| **Complement C3 (mg/dl)** | 125.33±20.75 | 107.15±20.18 | 97.99±18.77 |
| **Complement C4 (mg/dl)** | 33.17±8.88 | 26.75±7.06 | 26.62±7.87 |
| **IgA (mg/dl)** | 288.46±95.36 | 207.87±73.28 | 215.15±76.87 |
| **IgE (IU/ml)** | 158.91±258.65 | 279.85±881.37 | 71.98±65.33 |
| **IgG (mg/dl)** | 1428.57±299.93 | 1210.77±214.59 | 1199.14±265.93 |
| **IgM (mg/dl)** | 81.21±41.75 | 99.73±51.17 | 81.91±30.95 |
| **Ig light chain κ (mg/dl)** | 337.86±81.70 | 269.35±64.68 | 267.54±48.91 |
| **Ig light chain λ (mg/dl)** | 205.79±47.44 | 153.68±34.33 | 160.88±37.44 |
| **β2-microglobulin (mg/dl)** | 0.26±0.06 | 0.24±0.04 | 0.23±0.04 |
| **Prealbumin (mg/dl)** | 19.77±9.19 | 24.29±4.79 | 25.89±5.70 |
| **Transferrin (mg/dl)** | 177.68±27.97 | 210.92±34.88 | 218.00±42.84 |
| **Ceruloplasmin (mg/dl)** | 34.30±7.97 | 25.36±4.88 | 26.53±4.22 |
| **α1-acid glycoprotein (mg/dl)** | 134.58±41.30 | 77.80±27.31 | 72.92±16.28 |
| **Haptoglobin (mg/dl)** | 245.08±99.00 | 108.22±52.37 | 106.28±41.41 |
| **β1-globulin (%)** | 5.98±0.65 | 5.63±0.82 | 5.73±0.71 |
| **β2-globulin (%)** | 5.51±1.03 | 4.73±0.98 | 4.56±0.67 |
| **ALB (%）** | 52.45±4.70 | 59.88±4.14 | 61.35±2.62 |
| **α1-globulin (%)** | 5.71±1.80 | 3.84±0.67 | 3.42±0.58 |
| **α2-globulin (%)** | 11.34±2.02 | 8.90±1.41 | 8.81±0.88 |
| **γ-globulin (%)** | 19.00±3.23 | 17.02±2.49 | 16.13±2.51 |

**Supplementary Table 3. Accuracy of individual immune parameter in diagnosing periprosthetic joint infection.** AUC, area under the curve

|  | **AUC (95% CI)** | **Cutoff** | **Sensitivity** | **Specificity** |
| --- | --- | --- | --- | --- |
| **Percentage of NK cell** | 0.680(0.536-0.823) | 12.90 | 0.714 | 0.615 |
| **Absolute NK cell counts** | 0.657(0.510-0.803) | 179.0 | 0.385 | 0.893 |
| **Percentage of B cell** | 0.713(0.571-0.855) | 10.73 | 0.923 | 0.424 |
| **Absolute B cell counts** | 0.735(0.590-0.880) | 200.1 | 0.923 | 0.643 |
| **Complement C3** | 0.725(0.591-0.860) | 125.5 | 0.923 | 0.464 |
| **Complement C4** | 0.725(0.587-0.862) | 29.25 | 0.731 | 0.529 |
| **IgA** | 0.740(0.608-0.871) | 217.5 | 0.615 | 0.786 |
| **IgG** | 0.718(0.578-0.858) | 1265 | 0.692 | 0.750 |
| **Ig light chain κ** | 0.734(0.599-0.868) | 260.5 | 0.539 | 0.893 |
| **Ig light chain λ** | 0.805(0.690-0.920) | 172.5 | 0.808 | 0.714 |
| **Prealbumin** | 0.749(0.607-0.891) | 18.35 | 0.923 | 0.643 |
| **Transferrin** | 0.756(0.630-0.883) | 186.5 | 0.769 | 0.607 |
| **Ceruloplasmin** | 0.859(0.737-0.960) | 27.05 | 0.731 | 0.893 |
| **α1-acid glycoprotein** | 0.889(0.798-0.980) | 93.95 | 0.885 | 0.821 |
| **Haptoglobin** | 0.890(0.903-0.978) | 186.5 | 1.000 | 0.750 |
| **β2-globulin** | 0.697(0.557-0.837) | 5.950 | 0.923 | 0.429 |
| **ALB** | 0.889(0.803-0.975) | 57.95 | 0.731 | 0.929 |
| **α1-globulin** | 0.879(0.787-0.971) | 4.500 | 0.846 | 0.786 |
| **α2-globulin** | 0.852(0.745-0.958) | 10.00 | 0.885 | 0.786 |
| **γ-globulin** | 0.675(0.532-0.819) | 19.65 | 0.923 | 0.429 |
